# Highly efficient synthesis of arbutin esters catalyzed by whole cells of *Candida parapsilosis*†

**DOI:** 10.1039/c8ra00595h

**Published:** 2018-03-13

**Authors:** Xiaofeng Li, Haixia Xu, Guanglei Zhao, Hui Wu, Yigang Yu, Furao Lai, Xinglong Xiao

**Affiliations:** State Key Laboratory of Pulp and Paper Engineering, South China University of Technology Wushan Road 381 Guangzhou 510640 China glzhao@scut.edu.cn +86-20-87111770; School of Food Science and Engineering, South China University of Technology Guangzhou 510640 China fexxl@scut.edu.cn

## Abstract

Acylation modification of phenol glycosides is currently of great interest due to the improved bioavailability and multiple functions. In this work, mono- or diesters of arbutin, an important phenol glycoside derivative, can be controllably synthesized by using whole-cell biocatalytic systems. Among fourteen microbial strains selected, *Candida parapsilosis* cells showed the best catalytic activity and high organic solvent tolerance. Compared with the best pure solvent tetrahydrofuran, the use of a binary solvent pyridine-isooctane gave a slightly lower conversion (98.3% *vs.* 97.2%) and selectivity (85.3% *vs.* 80.5%) and much higher substrate solubility (37.1 *vs.* 214.0 mg mL^−1^), in a 24 h bioconversion of arbutin with a VP-arbutin molar ratio of 15 and whole cell dosage of 30 mg mL^−1^. The production of various arbutin esters with different fatty acid chain lengths can be realized by using this whole-cell strategy, with the substrate conversion and 6′-regioselectivity of 54.1–98.3% and 83.2–99.0%, respectively.

## Abstract 2

Acylation modification of phenol glycosides is currently of great interest due to the improved bioavailability and multiple functions.

## Graphical abstract

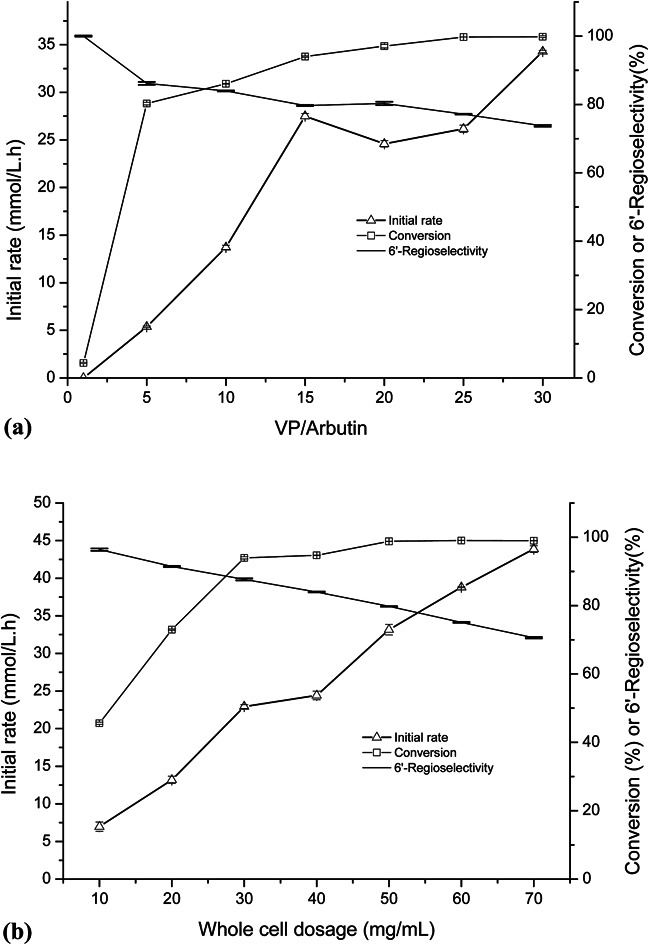

## 1. Introduction

Arbutin (hydroquinone-*o*-β-d-glucopyranosid, a natural phenol glycoside, is ubiquitous in edible plants such as strawberry plants,^1,2^*Bergenia crassifolia*^3^ and pear trees.^4^ Over the last decades, it has attracted significant attention due to its versatile biological activities which include anti-inflammatory treatment,^5^ scavenging free radicals^6^ and lowering blood sugar.^7^ Furthermore, it is well-known for its high tyrosinase-inhibiting activity through direct combination with tyrosinase, inhibiting the formation of melanin and accelerating its decomposition. This activity has found considerable applications in the cosmetic industry as a powerful vegetable-based whitening agent.^8,9^ However, arbutin suffers from low bioavailability due to its poor cell membrane penetration. Recently, many studies have indicated that acylation modification of arbutin could increase its antimelanogenesis and antioxidant activities. Nagai *et al.*^10^ investigated the antioxidative effects of various acylated arbutins and found that the oxidation of methyl linoleate was inhibited by the arbutin esters. Tokiwa *et al.*^11^ reported a much higher inhibitory effect of arbutin esters on mushroom tyrosinase than unmodified arbutin and indicated that the esters mainly inhibited the latter part of the tyrosinase catalyzed reaction.

Generally, ester derivatives of phenol glycoside are prepared chemically or enzymatically.^12^ Chemical acylation methods require complex procedures with multiple protection/deprotection steps, consuming a large amount of reagents, resulting in adverse environmental effects. Enzymatic acylation is superior to commonly used chemical approaches considering its high regioselectivity, mild reaction conditions, and environmental friendliness.^13,14^ However, these enzymatic methods suffer from the high cost, low stability, and the lack of enzyme recyclability. Thus, great effort has been devoted to screening for low-cost enzymes, development of enzyme immobilization strategies, and searching for new enzymes with high stability.^11,15–17^

**Scheme 1:**
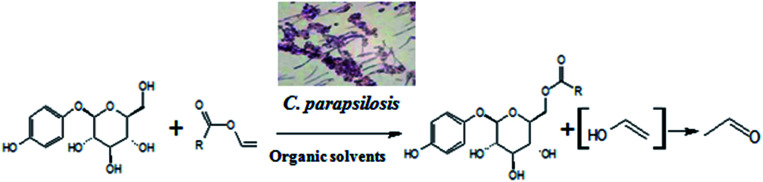
Regioselective acylation of arbutin by microbial whole cells.

Whole cells are another kind of biocatalyst, different from pure enzymes, and exert catalytic functions based on the cell-bound/endoenzymes. This kind of biocatalyst has the advantages inherent in enzymatic processes, and has its own advantages,^18–21^ including higher stability compared to pure enzymes,^22^ coenzyme regeneration for multi-step biotransformation reaction,^23^*etc.* To the best of our knowledge, no studies have been reported on whole-cell-catalyzed acylation of arbutin. Herein, we report, for the first time, a facile and highly efficient biocatalytic method for the acylation of arbutin with vinyl propionate (VP) using microbial whole-cells (Scheme 1). The origin of the cells and solvent effects on the reaction were investigated. In addition, the established biocatalytic process was used for the synthesis of arbutin esters with different fatty acid chain lengths.

## 2. Methods and materials

### 2.1 Biological and chemical materials

Arbutin was purchased from Aladdin. *Rhizomucor miehei* GIM 3.510, *Rhizopus chinensis* GIM 3.144, *Mucor circinelloides* GIM 3.521, *Rhizopus oryzae* GIM 3.509, *Aspergillus niger* GIM 3.25, *Rhizopus oligosporus* GIM 3.515, *Candida parapsilosis* GIM 2.190, *Pseudomonas putida* GIM 1.193, *Bacillus subtilis* GIM 1.135, *Pseudomonas fluorescent* GIM 1.209, *Pseudomonas cepacia* GIM 1.139, *Pseudomonas stutzeri* GIM 1.273, *Aspergillus oryzae* GIM 3.5232, *Pseudomonas aeruginosa* GIM 1.46, *Geotrichum candidum* GIM 2.12 were supplied by the Guangdong Culture Collect Centre. The acyl donors all were purchased from TCI (Japan). All other chemicals were from commercial sources and were of the highest purity available.

### 2.2 Preparation of whole-cell biocatalysts

The bacterial and yeast strains were activated using a nutrient broth medium at 37 °C and 180 rpm for 24 h. Fungal strains were activated with a potato-dextrose-agar culture medium at 28 °C for 60 h. After activation, 5% (v/v) seed cultures were inoculated into the culture medium containing 5.0 g L^−1^ yeast extract, 5.0 g L^−1^ (NH_4_)_2_SO_4_, 1.0 g L^−1^ K_2_HPO_4_, 0.2 g L^−1^ MgSO_4_·7H_2_O, and 5.0 g L^−1^ soybean oil. The cultivation was carried out on a rotary shaker at 37 °C and 180 rpm for 48 h. Then the bacterial and yeast cells were collected by centrifugation (10 000 rpm, 3 min) and the supernatant fractions were discarded. For the fungal stains, the cells were collected by vacuum filtration. Then the cells achieved were washed twice with distilled water to remove the residual medium, freeze-dried at −45 °C for 24 h and stored at 4 °C.

### 2.3 General procedure for whole cell mediated acylation of arbutin

In a typical experiment, arbutin (20 mM L^−1^), VP (900 mM L^−1^) and freeze-dried cells (40 mg mL^−1^) were added to 2 mL of anhydrous organic solvents and the mixture incubated at 40 °C in an orbital air-bath shaker (200 rpm). Aliquots were took at specified time intervals from the reaction mixture, the whole-cell catalyst was separated by centrifugation (12 000 rpm, 5 min), then taking 40 μL of supernatant diluted with chromatographic grade methanol 25-fold for HPLC detection with a 20 μL sample injection volume. The initial rate was calculated according to the reduction of substrate in the initial stage of 1 hour. Regioselectivity was defined as the molar ratio of the desired product to the total amount of ester products formed. All data are averages of at least two separate experiments.

### 2.4 Operational stability

A total of 2 mL of anhydrous THF containing arbutin (20 mM L^−1^), VP (300 mML^−1^) and the whole-cell catalyst (30 mg mL^−1^) incubated at 40 °C in an orbital air-bath shaker (200 rpm). After 24 h, the whole-cell catalyst was separated by centrifugation (12 000 rpm, 5 min), then taking 40 μL of supernatant diluted with chromatographic grade methanol 25-fold for HPLC detection with a 20 μL sample injection volume. After 24 h of reaction, the rest of the whole-cell catalyst was washed with anhydrous THF three times, and dried for 2 h at 40 °C. This regenerated the whole-cell catalyst for use in the next catalytic reaction batch, and this process was repeated 6 times.

### 2.5 HPLC analysis

The reaction mixture was analyzed by RP-HPLC on a 4.6 mm × 250 mm (5 μm) Zorbax SB-C18 column (Agilent Technologies Industries Co., Ltd., USA) using a Waters 600 Four element pump and a UV detector at 283 nm. The mobile phase is a mixture of methanol and water at 0.9 mL min^−1^. The volumetric ratio of methanol and water and the retention times for arbutin and its 6′-*O*-monoester were 40/60, 3.063 and 3.279 min (acetylation), 55/45, 3.080 and 3.740 min (propionylation), 70/30, 3.075 and 7.548 min (octanoylation), 80/20, 3.066 and 10.751 min (lauroylation), 3.066 and 6.334 min (10-undecenoylation), 85/15, 3.055 and 7.302 min (myristoylation), 90/10, 3.044 and 6.334 min (palmitoylation), 95/5, 3.054 and 10.809 min (stearylation), respectively.

### 2.6 Separation and structural characterization of the products

After the reaction, the whole-cell catalyst was separated by centrifugation, and the supernatant was evaporated under vacuum to remove solvent. The residue was isolated to different component with thin-layer chromatography (TLC) method using chloroform/methanol as mobile phase. After crystallization under vacuum drying, the powder of products were obtained. The acylation position of the products were determined by ^13^C NMR (Bruker AVANCE Digital 400 MHz Nuclear Magnetic Resonance Spectrometer, Bruker Co., Germany) at 100 MHz. DMSO-*d*_6_ was used to dissolve substrate and product, the ppm shifts indicate chemical shifts. The mass spectra of products were obtained on Bruker maXis impact high-resolution mass spectrometry.

#### Arbutin

^13^C NMR (DMSO-d_6_, 100 MHz): *δ* ppm 152.67 (C4), 150.86 (C1), 118.21 (C2, C6), 115.99 (C3, C5), 102.26 (C1′), 77.42 (C3′), 77.13 (C5′), 73.80 (C2′), 70.34 (C4′), 61.34 (C6′).

#### Arbutin-6′-acetate

^13^C NMR (DMSO-d_6_, 100 MHz): *δ* ppm 170.66 (C1′′), 152.29 (C4), 150.57 (C1), 118.16 (C2, C6), 115.97 (C3, C5), 101.95 (C1′), 76.85 (C3′), 74.29 (C5′), 74.01 (C2′), 70.44 (C4′), 63.94 (C6′), 21.10 (C2′′). HRMS (*m*/*z*): 337.09 (M + Na)^+^.

#### Arbutin-6′-propionate

^13^C NMR (DMSO-d_6_, 100 MHz): *δ* ppm 173.91 (C1′′), 152.84 (C4), 150.56 (C1), 118.12 (C2, C6), 115.93 (C3, C5), 101.95 (C1′), 76.87 (C3′), 74.11 (C5′), 73.70 (C2′), 70.55 (C4′), 63.94 (C6′), 27.35 (C2′′), 9.44 (C3′′). HRMS (*m*/*z*): 351.11 (M + Na)^+^.

#### Arbutin-3′,6′-dipropionate

^13^C NMR (DMSO-d_6_, 100 MHz): *δ* ppm 173.57 × 2(C1′′), 153.18 (C4), 150.23 (C1), 118.25 (C2, C6), 116.01 (C3, C5), 101.99 (C1′), 74.15 (C3′), 74.15 (C5′), 73.71 (C2′), 71.66 (C4′), 63.94 (C6′), 27.27 × 2 (C2′′), 9.42 (C3′′). HRMS (*m*/*z*): 407.13 (M + Na)^+^.

#### Arbutin-6′-caprylate

^13^C NMR (DMSO-d_6_, 100 MHz): *δ* ppm 173.20 (C1′′), 152.85 (C4), 150.55 (C1), 118.08 (C2, C6), 115.90 (C3, C5), 101.97 (C1′), 76.85 (C3′), 74.09 (C5′), 73.66 (C2′), 70.54 (C4′), 63.86 (C6′), 34.01 (C2′′), 31.54 (C6′′), 28.87 (C5′′), 28.78 (C4′′), 24.89 (C3′′), 22.48 (C7′′), 14.38 (C8′′). HRMS (*m*/*z*): 421.18 (M + Na)^+^.

#### Arbutin-6′-undecenoate

^13^C NMR (DMSO-d_6_, 100 MHz): *δ* ppm 173.23 (C1′′), 152.82 (C4), 150.56 (C1), 139.29 (C10′′), 118.10 (C2, C6), 115.90 (C3, C5), 115.06 (C11′′), 101.97 (C1′), 76.81 (C3′), 74.09 (C5′), 73.65 (C2′), 70.53 (C4′), 63.85 (C6′), 34.00 (C2′′), 33.61 (C9′′), 29.12 (C4′′), 28.90 (C5′′), 28.89 (C6′′, C7′′), 28.70 (C8′′), 24.86 (C3′′). HRMS (*m*/*z*): 461.38 (M + Na)^+^.

#### Arbutin-6′-laurate

^13^C NMR (DMSO-d_6_, 100 MHz): *δ* ppm 13.07(C12′′), 22.34 (C11′′), 24.72 (C3′′), 28.87 (C4′′), 28.90 (C5, C9′′), 29.20 (C8′′), 29.88 (C6′′, C7′′), 31.67 (C10′′), 33.71 (C2′′), 63.53 (C6′), 70.41 (C4′), 73.53 (C2′), 73.69 (C5′), 76.52 (C3′), 102.30 (C1′), 115.22 (C3, C5), 118.27 (C2, C6), 150.86 (C1), 152.60 (C4), 173.92 (C1′′). HRMS (*m*/*z*): 477.24 (M + Na)^+^.

#### Arbutin-6′-myristate

^13^C NMR (DMSO-d_6_, 100 MHz): *δ* ppm 173.21 (C1′′), 152.84 (C4), 150.56 (C1), 118.09 (C2, C6), 115.89 (C3, C5), 101.99 (C1′), 76.83 (C3′), 74.09 (C5′), 73.65 (C2′), 70.53 (C4′), 63.87 (C6′), 34.00 (C2′′), 31.74 (C12′′), 29.49 (C6′′, C7′′, C8′′), 28.91 (C9′′), 28.90 (C5′′, C11′′), 28.84 (C4′′), 24.87 (C3′′), 22.54 (C13′′), 14.40 (C14′′). HRMS (*m*/*z*): 505.28 (M + Na)^+^.

#### Arbutin-6′-palmitate

^13^C NMR (DMSO-d_6_, 100 MHz): *δ* ppm 14.37 (C16′′), 22.52 (C15′′), 24.86 (C3′′), 28.22 (C4′′), 28.52 (C5′′), 28.90 (C13′′), 29.20 (C6′′, C7′′, C8′′, C9′′, C10′′, C11′′, C12′′), 31.83 (C14′′), 34.09 (C2′′), 63.86 (C6′), 70.55 (C4′), 73.66 (C2′), 74.10 (C5′), 76.83 (C3′), 102.01 (C1′), 115.90 (C3, C5), 118.12 (C2, C6), 150.57 (C1), 152.84 (C4), 173.22 (C1′′). HRMS (*m*/*z*): 533.31 (M + Na)^+^.

#### Arbutin-6′-stearate

^13^C NMR (DMSO-d_6_, 100 MHz): *δ* ppm 14.37 (C18′′), 22.52 (C17′′), 24.86 (C3′′), 28.52 (C4′′), 29.18 (C5′′), 29.53 (C15′′), 29.55 (C6′′, C7′′, C8′′, C9′′, C10′′, C11′′, C12′′, C13′′, C14′′), 31.72 (C16′′), 33.79 (C2′′), 63.85 (C6′), 70.54 (C4′), 73.66 (C2′), 74.10 (C5′), 76.82 (C4′), 102.00 (C1′), 115.90 (C3, C5), 118.12 (C2, C6), 150.57 (C1), 152.81 (C4), 173.23 (C1′′). HRMS (*m*/*z*): 561.34 (M + Na)^+^.

### 2.7 Determination of the log *p* values of the ester derivatives of arbutin

The 1-octanol-water partition coefficients (log *p*) of the acylated derivatives were measured according to a previously published method.^24^ Each derivative was dissolved in 1-octanol (100 μM, 2 mL) and then mixed vigorously with 2 mL of water. After equilibration for 10 min, the derivatives in each layer were measured and their amounts were assessed by using the HPLC analytical process. The log *p* was calculated by taking the logarithm of the ratio of the HPLC peak area of each acylated derivative in octanol to the corresponding peak area in water.

### 2.8 Determination of arbutin solubility

To determine its solubility, 20 mg of arbutin standard was dissolved in 1 mL of methanol to prepare a 20 mg mL^−1^ standard stock solution. A 50 μL standard storage solution was diluted to 1000 μg mL^−1^, and 0.1, 0.2, 0.4, 0.6, 0.8, and 1 mL of the standard solution (1000 μg mL^−1^) were diluted to 100, 200, 400, 600, 1000 μg mL^−1^, respectively, and a sample volume of 20 μL was used for HPLC detection.

Arbutin saturated solution configuration: to 1 mL of organic solvent, an excess of arbutin was added. After mixing at 12 000 rpm in a centrifuge for 3 min, 20 μL of the supernatant was used for HPLC detection, and its solubility was calculated.

## 3 Results and discussions

### 3.1 Catalytic behaviors of different strains for arbutin acylation

Table 1 shows the catalytic behaviors of seven bacterial strains, six fungal strains and one yeast strain possessing lipase activity. All the fungal strains tested (entries 1–7) showed the catalytic ability towards catalysis of arbutin propionylation. Whole-cell of *Rhizomucor miehei* showed the highest activity with a substrate conversion up to 27.2%. Among the six bacterial strains (entries 8–13), only *Pseudomonas putida* and *Bacillus subtilis* were able to catalyze the reaction with the conversion rate being of 39.8% and 8.0%, respectively. Among the fourteen strains tested, *Candida parapsilosis* (entry 14) showed the best catalytic activity, and the conversion rate reached 98.3% after 24 h.

**Table 1:** Transesterification of arbutin with VP by whole-cell catalysts

Entry	Strains	*V*_0_ (mM h^−1^ L^−1^)	Conversion^a^ (%)	6′-Regioselectivity (%)
1	*Rhizomucor miehei* GIM 3.510	3.9 ± 0.2	27.2 ± 0.1	>99.0
2	*Rhizopus chinensis* GIM 3.144	2.5 ± 0.0	22.2 ± 0.5	>99.0
3	*Mucor circinelloides* GIM 3.521	NA	15.5 ± 0.5	>99.0
4	*Rhizopus oryzae* GIM 3.509	1.5 ± 0.3	13.6 ± 0.6	>99.0
5	*Aspergillus niger* GIM 3.25	NA	6.5 ± 0.3	>99.0
6	*Rhizopus oligosporus* GIM 3.515	NA	5.9 ± 0.7	>99.0
7	*Geotrichum candidum* GIM 2.12	NA	NA	NA
8	*Pseudomonas putida* GIM 1.193	1.8 ± 0.1	39.8 ± 0.4	>99.0
9	*Bacillus subtilis* GIM 1.135	NA	8.0 ± 0.3	>99.0
10	*Pseudomonas fluorescent* GIM 1.209	NA	NA	NA
11	*Pseudomonas cepacia* GIM 1.139	NA	NA	NA
12	*Pseudomonas stutzeri* GIM 1.273	NA	NA	NA
13	*Pseudomonas aeruginosa* GIM 1.46	NA	NA	NA
14	*Candida parapsilosis* GIM 2.190	23.7 ± 0.3	98.3 ± 0.1	85.3 ± 0.9

a The reactions were carried out in THF (2 mL) with arbutin (20 mM L^−1^), VP (900 mM L^−1^) and whole-cell catalysts (40 mg mL^−1^) at 40 °C and 200 rpm.

The products were separated with thin-layer chromatography (TLC) method and structural analyzed by HRMS and ^13^C NMR spectrums. HRMS analysis of the separated products showed that only two kinds of esters produced in the acylation process. The acylation positions of acylated products were further confirmed based on the significant chemical shift differences between arbutin and the ester products. Results showed that a mono-acylation occurred on the C6′ of sugar moiety of arbutin, which led to a downfield shift of the C6′ signal of 2.60 ppm and an upfield shift of the directly neighboring carbon atom C5′ signal of 2.84 ppm. In addition, the signal peak of C=O appeared at 173.91 ppm or 170.66 ppm. And the carbon atoms of the fatty acid chain also appeared at 27.35 ppm (–CH–) and 9.44 ppm (CH_3_). The ^13^C NMR spectrum of another kind of ester shows downfield shifts of two carbon atoms C3′ and C6′ (3.27 ppm and 2.98 ppm, respectively) and upfield chemical shifts of their neighboring carbon atoms C4′ and C5′ (1.32 ppm and 2.60 ppm, respectively). Besides, two signal peaks of C=O appeared at 173.91 ppm, confirming that 3′,6′-diester of arbutin was formed.

Interestingly, whole cells of *C. parapsilosis* catalyzed the reaction producing both mono- and di-esters, while the other molds and bacteria tested showed high 6′-regioselectivity (>99.0%) producing only monoesters. The results indicated that the cell-bound enzymes from various microbial sources have different substrate recognition characteristics.^25–27^

### 3.2 Effect of the reaction media

Generally speaking, organic solvents have a great impact on non-aqueous catalysis,^28^ affecting the stability and activity of the biocatalysts, and substrate solubility.

Table 2 (entries 1–5) shows the influence of different pure solvents on the whole-cell mediated acylation of arbutin. As a polar compound, arbutin is highly soluble in three polar pure solvents (DMSO, DMF, and pyridine). However, no cellular catalytic activity was tested in DMF and DMSO, which is likely because highly polar solvents deactivate the cell-bound enzymes by stripping off the essential water layer, causing protein aggregation and precipitation as well as “interfacial inactivation”.^29–31^ Although high initial production rates and conversions were observed in solvents with lower polarity, the solubility of arbutin was lower than 37 mg mL^−1^. To increase the solubility of arbutin in hydrophobic organic solvents and improve the reaction efficiency, a series of binary solvent mixtures containing the polar solvent pyridine and various hydrophobic solvents (1 : 1, v/v) were tested as reaction media for the whole-cell *Candida parapsilosis* catalyzed transesterification of arbutin with VP. As shown in Table 2 (entries 6–10), compared with the pure solvents system of THF and *t*-pentanol, the solubility of arbutin in their corresponding binary solvents was greatly improved, 4–5 times higher than in the pure hydrophobic solvents. In pyridine/acetone, both the initial rate and conversion of the reaction were much higher than pure pyridine. This may be due to the addition of hydrophobic organic solvents reducing the polarity of the reaction system, thereby reducing the toxic effects on the cells and the cell-bound lipases.^32^ Among the five binary solvents tested, pyridine-isooctane gave the best initial rate (37.2 mM h^−1^ L^−1^) and substrate conversion (97.2%), similar to those achieved in pure THF. The results indicate that a suitable binary solvent (pyridine-isooctane) can be used instead of the pure solvents to achieve a highly efficient reaction with a greatly increased substrate concentration. Interestingly, 6′-regioselectivity was negatively correlated with conversion yield in pure solvents, consistent with the report of Li *et al.*^33^ Herein, *Candida parapsilosis* cells exhibited relatively high organic solvent tolerance, which can be used as a promising catalysts for non-aqueous reaction.

**Table 2:** Effect of organic solvents on *C. parapsilosis* whole cell catalyzed acylation of arbutin

Entry	Solvents^a^	Solubility (mg mL^−1^)	*V*_0_ (mM h^−1^ L^−1^)	Conversion (%)	6′-Regioselectivity (%)
1	DMF	275.9 ± 0.1	NA^b^	NA	NA
2	DMSO	227.0 ± 0.3	NA	NA	NA
3	Pyridine	192.6 ± 0.5	6.5 ± 0.2	42.9 ± 0.9	96.1 ± 0.8
4	THF	37.1 ± 0.5	23.7 ± 0.3	98.3 ± 0.1	85.3 ± 0.9
5	*t*-Pentanol	35.2 ± 0.2	15.1 ± 0.3	89.3 ± 0.2	87.7 ± 0.9
6	Pyridine/*t*-butanol	134.0 ± 0.7	16.6 ± 0.1	86.5 ± 0.3	92.9 ± 0.7
7	Pyridine/*t*-pentanol	160.3 ± 0.4	12.6 ± 0.0	88.8 ± 0.4	89.1 ± 1.0
8	Pyridine/acetone	163.0 ± 0.5	5.6 ± 0.2	89.1 ± 0.4	86.2 ± 0.3
9	Pyridine/THF	191.8 ± 0.6	19.7 ± 0.2	85.6 ± 0.5	90.0 ± 0.3
10	Pyridine/isooctane	214.0 ± 0.3	37.2 ± 0.0	97.2 ± 0.5	80.5 ± 0.4

a The reaction conditions for pure solvents: arbutin 20 mM L^−1^, VP 900 mM L^−1^ and whole-cells 40 mg mL^−1^, 40 °C and 200 rpm; the reactions for binary solvents: solvent ratio 1 : 1 (v/v), arbutin 20 mM L^−1^, VP 150 mM L^−1^, whole-cells 30 mg mL^−1^, 40 °C and 180 rpm.

b No activity.

### 3.3 Effects of reaction conditions on the whole-cell catalyzed acylation of arbutin

**Fig. 1:**
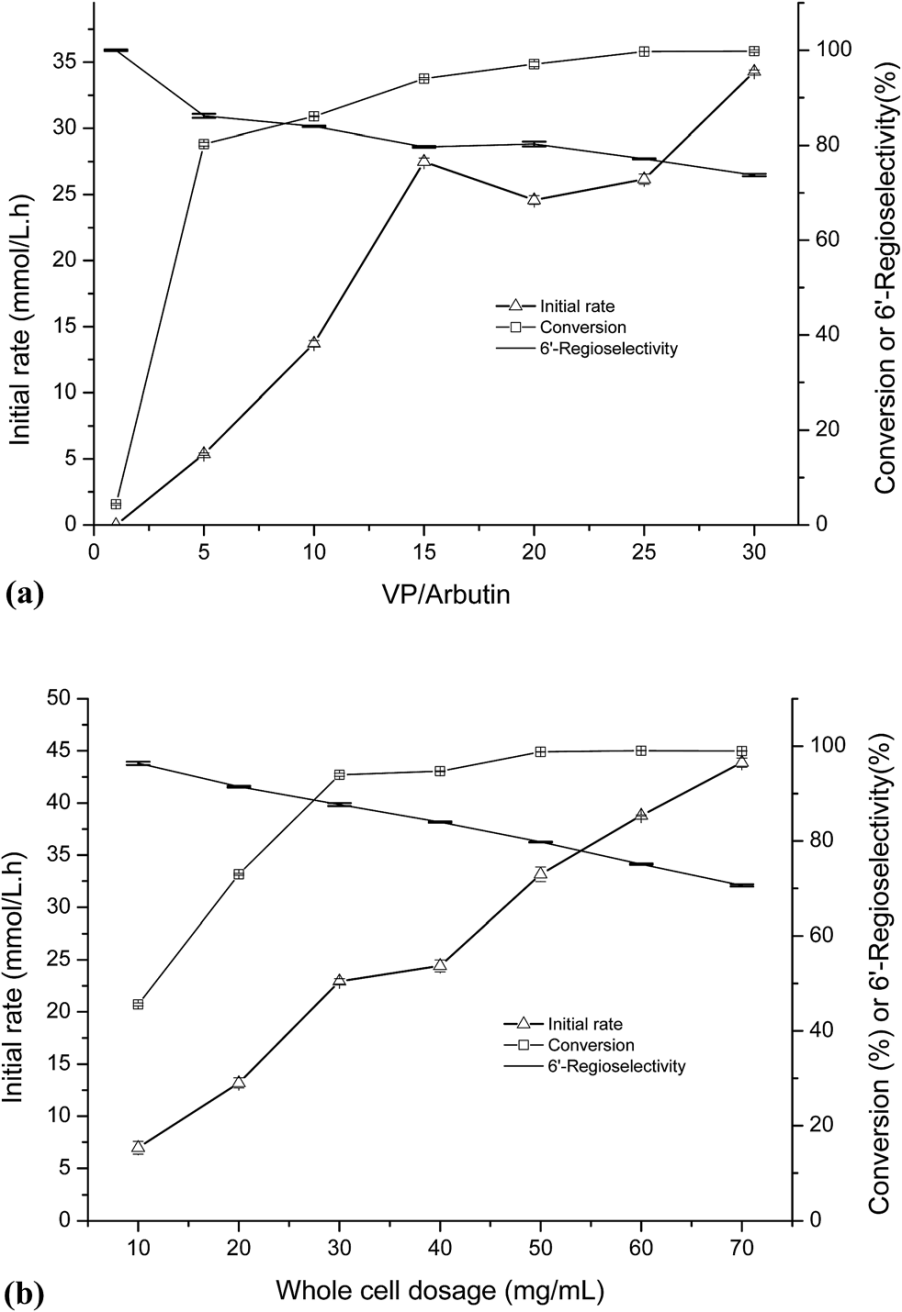
(a) Effect of molar of vinyl propionate on *C. parapsilosis* whole cell catalyzed acylation of arbutin. The reactions were carried out in THF (2 mL) with arbutin (30 mM L^−1^), VP and whole-cell catalysts (40 mg mL^−1^) at 40 °C and 200 rpm. (b) Effect of *C. parapsilosis* whole cell dosage on arbutin acylation. The reactions were carried out in THF (2 mL) with arbutin (30 mM L^−1^), VP (450 mg mL^−1^) and whole-cell catalysts at 40 °C and 200 rpm.

As shown in Fig. 1a, the initial rate of the reaction increased along with the increased of the VP/arbutin molar ratio. When the molar ratio of VP/arbutin increased from 0 to 5 mM mM^−1^, the substrate conversion increased sharply and then slowly until it reached above 90.0%. These results are consistent with those of Li *et al.*^33^ who found that increased molar ratio of substrates resulted in higher initial reaction rates and conversion yields of the enzymatic acylation of dihydromyricetin. The rate increased sharply, and further increases in the substrate molar ratio only resulted in small gains in reaction rate and conversion. The acylation of arbutin is a reversible reaction in non-aqueous media, accompanied by the hydrolysis of the enol ester (a side reaction that evidently consumes the acyl donor). Increasing the VP ratio may provide a sufficient amount of acyl donor and favor of the acylation of arbutin.^34^ However, the 6′-regioselectivity decreased with increasing of the VP/arbutin molar ratio, which may be due to the high acyl donor concentration results in an increase in the formation of di-esters. Higher acyl donor concentration promoted the formation of the acyl-enzyme intermediate and increased the rate of the nucleophilic attack of different alcohol ends (arbutin) on the carbonyl carbon of the intermediate.^35^ The result is similar to those reported by Chebil *et al.*^25^ who found that with an increasing molar ratio of vinyl acetate to quercetin, the proportion of the monoester decreased. In contrast, Li *et al.*^33^ reported that the molar ratio of acyl donor to dihydromyricetin showed only a minor effect on the proportion of dihydromyricetin-3-acetate in the total acylation products.

As shown in Fig. 1b, with increasing whole cell dosage, the initial rate gradually increased. The conversion rate increased more quickly when the whole cell dosage was increased from 20 to 30 mg mL^−1^ and then increased slowly until it reached a relatively constant value of 90% at whole-cell dosages above 50 mg mL^−1^. This is mainly because of the stronger mass-transfer limitations induced by the higher cell dosages. When the enzymes are saturated, increasing the amount of enzyme present has little influence on substrate conversion. Thus, to conserve the whole-cell catalyst, choosing a suitable whole cell dosage is necessary. Interestingly, the results show a trend towards decreasing the 6′-regioselectivity of the reaction with increasing whole cell dosage. According to classical enzymatic theory, this can be ascribed to the formation of arbutin monoester-enzyme intermediate promoted by higher whole-cell dosages, which results in increased production of the double ester.

### 3.4 Time course of the whole-cell catalyzed acylation of arbutin

**Fig. 2:**
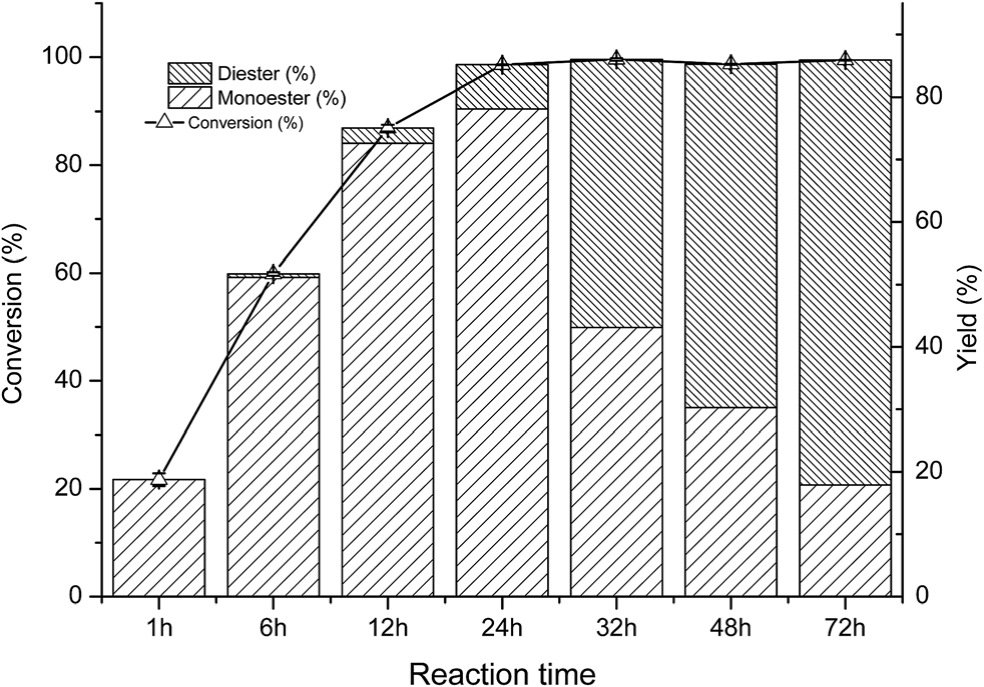
Time course of *C.parapsilosis* whole cell catalyzed acylation of arbutin. The reaction were carried out in THF (2 mL) with arbutin (20 mM L^−1^), VP (300 mM L^−1^) and whole-cell catalysts (30 mg mL^−1^) at 40 °C and 200 rpm.

As shown in Fig. 2, at the initial stage of the reaction, the conversion of arbutin was significantly increased, and the acylation reaction reached equilibrium after approximately 24 h. It is interesting to note that only the monoester was formed at the early stage of the reaction (0–1 h). After that, the yield of monoester increased while a small amount of the diester was produced and continuously increased. When the reaction time was prolonged greater than 24 h, the monoester content was reduced and the di-ester yield increased significantly. Similar results were obtained by Chebil *et al.*^25^ during the synthesis of isoquercitrin esters with a lipase PSL-C and Sin *et al.*^36^ achieved similar results during the synthesis of fructose esters with *Pseudomonas* sp.

**Fig. 3:**
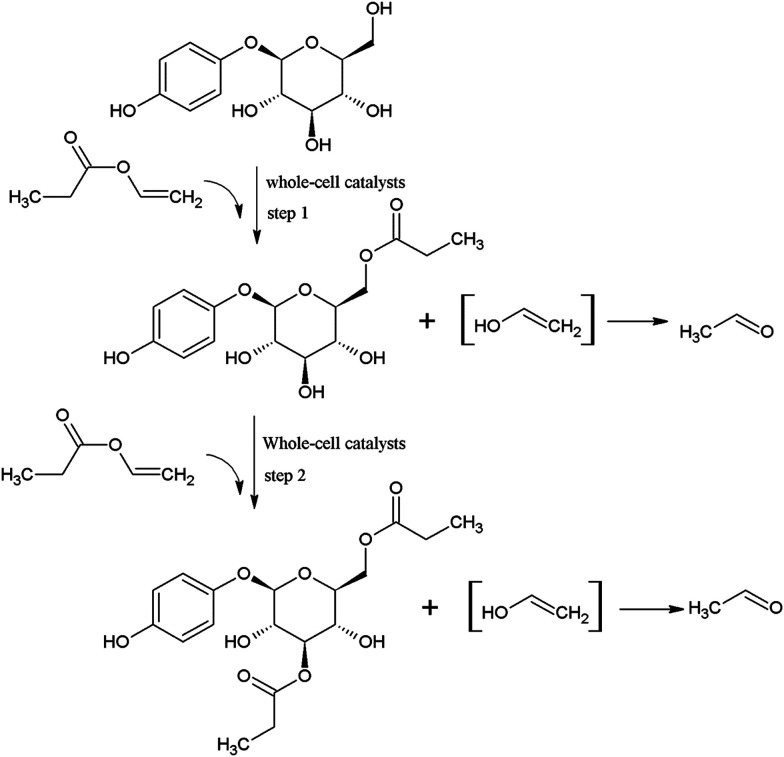
Schematic representation of synthesis process of arbutin esters.

Therefore, it is likely that the whole-cell catalyzed acylation of arbutin contained two sequential steps (Fig. 3). First, the primary hydroxyl group of arbutin is proposed to attack the acyl-enzyme intermediate to form the 6′-monoester of arbutin. Then, the 3′-hydroxyl group of the sugar moiety of the 6′-monoester attacks the acyl-enzyme intermediates, forming a 3′,6′-diester. This also explains the decrease in the 6′-regioselectivity, as shown in Fig. 1a and b, which increases the rate of step 2.

### 3.5 Regioselective acylation of arbutin with various acyl donors

In addition to the propionate acylation of arbutin, whole-cell biocatalysis was applied to the acylation of arbutin with acyl donors of differing fatty acid chain lengths. Several studies have shown that the long chain fatty acid esters of phenol glycosides have higher liposolubility and stronger bioactivities.^27^Table 3 shows that when the length of carbon chain was increased from 2 to 18, both the initial rate and substrate conversion significantly decreased. The increase in carbon chain length significantly enhanced the steric hindrance of the acyl donor molecules, preventing the formation of the acyl-enzyme intermediate and reducing the efficiency of the enzymatic reaction. For fatty acid vinyl esters with higher carbon-chain lengths (C3–C18), the conversion yield dropped constantly, and the whole-cell catalysis was most efficient for C3, with a conversion of 98.3% after 24 h. However, the 6′-regioselectivity of the reaction increased, which can be attributed to the steric hindrance of the reaction. With increasing fatty acid chain length of the acyl donors, the steric hindrance of the monoester increases, and preventing monoester attack of the acyl donor-enzyme intermediates to form the diester.

**Table 3:** Effect of fatty acid chain length of acyl donors on *Candida parapsilosis* catalyzed synthesis of arbutin esters^a^

Acyl donor	Structural formula of acyl donors	*V*_0_ (mM h^−1^ L^−1^)	Conversion (%)	6′-Regioselectivity (%)	log *p* of the monoester
Vinyl acetate	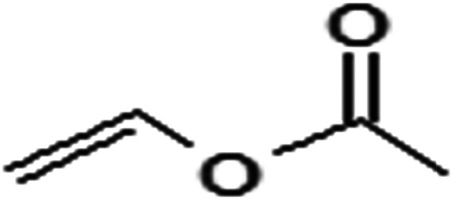	45.8 ± 0.3	97.6 ± 0.6	83.2 ± 0.3	−0.8 ± 0.1
Vinyl propionate	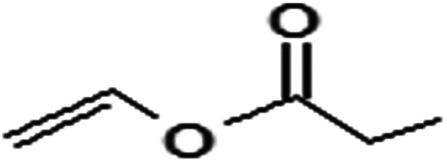	23.7 ± 0.3	98.3 ± 0.1	85.3 ± 0.9	−0.3 ± 0.3
Vinyl caprylate	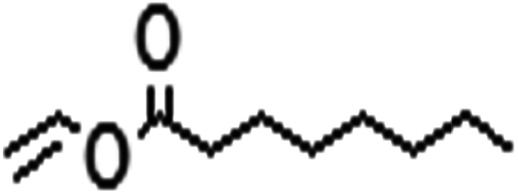	6.8 ± 0.9	92.4 ± 1.0	94.7 ± 0.7	2.1 ± 0.3
Vinyl 10-undecenoate	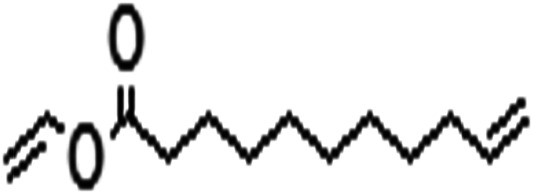	4.7 ± 0.2	90.3 ± 0.3	>99.0	2.2 ± 0.5
Vinyl laurate	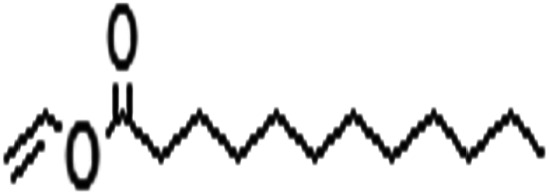	3.0 ± 0.5	71.1 ± 0.5	>99.0	2.4 ± 0.7
Vinyl myristate	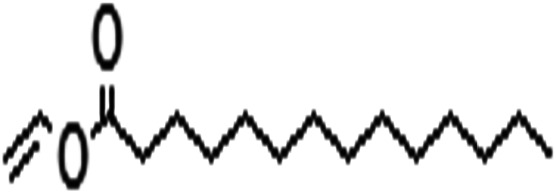	3.8 ± 0.1	69.7 ± 0.3	>99.0	4.0 ± 0.9
Vinyl palmitate	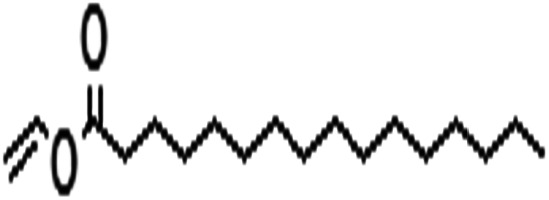	2.5 ± 0.7	55.9 ± 0.1	>99.0	5.0 ± 0.4
Vinyl stearate	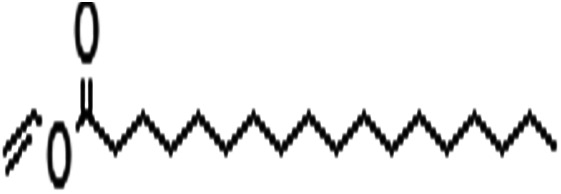	7.7 ± 0.9	54.1 ± 0.1	>99.0	5.3 ± 0.3

a The reactions were carried out in THF (1 mL) with arbutin (20 mM L^−1^), VP (900 mM L^−1^) and whole-cell catalysts (40 mg mL^−1^) at 40 °C and 200 rpm.

The log *p* value is a common parameter reflecting the liposolubility of a compound determined by its distribution in oil and water phases.^37^Table 3 shows that when the fatty acid chain length of the acyl donor increased, the log *p* values of the acylated derivatives increased gradually from −0.8 to 5.3. This suggests that the enzymatic acylation could effectively improve the lipophilicity of the hydrophilic arbutin. It also indicates that the acylation of phenol glycoside is an effective method of increasing liposolubility and several studies have reported that acylated derivatives have higher activity.^38–40^

### 3.6 Operational stability of the whole cell biocatalyst

**Fig. 4:**
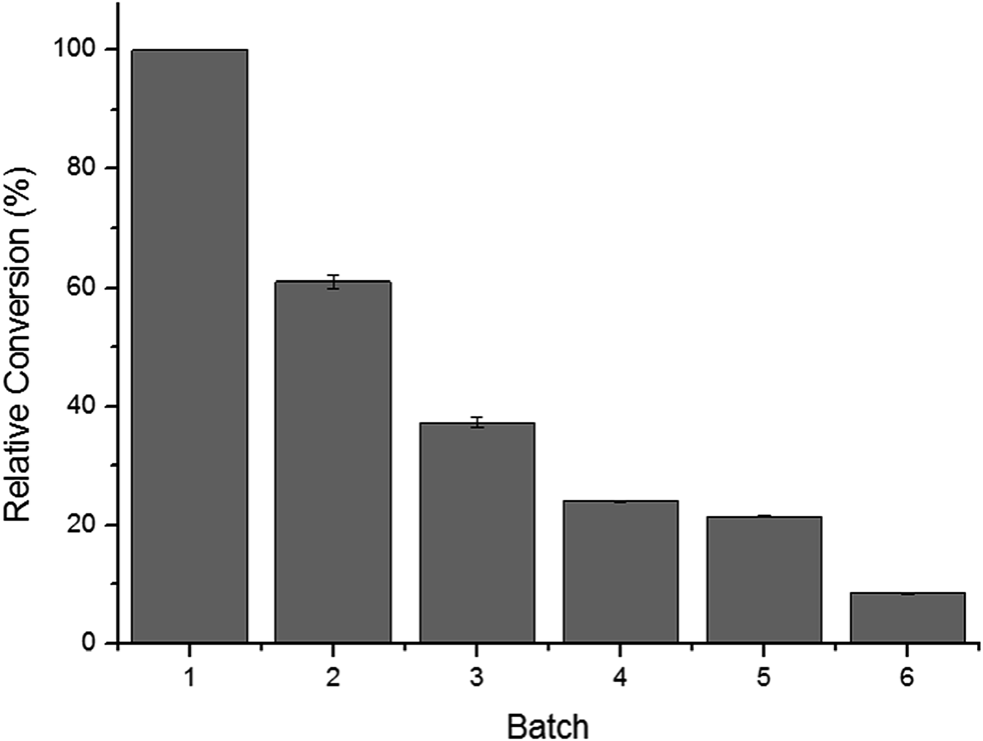
Operational stability of *C. parapsilosis* whole-cell catalyst.

Fig. 4 shows the operational stability of the whole-cell biocatalysts. It was observed that the cells retained high catalytic activity in their second use. After repeated used (three cycles), the activity of the whole cells was reduced to nearly 60%. In the fourth cycle, the conversion rate decreased rapidly below 40%, likely due to the toxic effects of the organic solvents to the cell structures and cell-bound enzymes. One of the major shortcomings of free enzymes is that they can only be used once. Thus, great effort has been made on the development of immobilization materials and strategies for enzymes. The results presented herein demonstrated that *C. parapsilosis* whole-cell catalysts are advantageous over free enzymes in their operational stability. The cell structure may serve as a natural carrier for loading and protecting enzymes, avoiding further immobilization and lowering the total costs of processing.

## 4. Conclusions

This study details a facile and efficient biocatalytic method for the synthesis of arbutin esters using *C. parapsilosis* whole-cells as a catalyst. With this approach, high yield and high regioselectivity of 6′-monoesters of arbutin were obtained within 24 h, while high yields of diesters can be obtained by prolonging the reaction to greater than 72 h. The catalytic performance and operational stability of the whole-cells for the acylation of arbutin may provide new opportunities to develop green industrial processes for novel phenol glycoside derivative production. Investigations on the bioactivities of the ester products and a comparative study of the phenol glycoside compounds with similiar structures to arbutin on the reactions are in progress.

## Conflicts of interest

There are no conflicts to declare.

## Supplementary Material

RA-008-C8RA00595H-s001
