# Supplementary material for: Highly efficient synthesis of arbutin esters catalyzed by whole cells of Candida parapsilosis
Source: RSC Adv. 2018 Mar 13;8(18):10081–8. doi: 10.1039/c8ra00595h (PMC9078728; doi:10.1039/c8ra00595h)
Supplement: RA-008-C8RA00595H-s001 [file RA-008-C8RA00595H-s001.pdf]

Electronic Supplementary Material (ESI) for RSC Advances.
This journal is © The Royal Society of Chemistry 2018

Highly efficient synthesis of arbutin esters catalyzed by whole cells of *Candida parapsilosis*

All authors and their affiliations:

Dr. Xiaofeng Li, first author

School of Food Sciences and Engineering, South China University of Technology, Wushan Road 381, Guangzhou 510641, China

Tel: (+86)20-22236819; E-mail: xflibio@scut.edu.cn ; Fax: (+86)20-87112853

Haixia Xu,

School of Food Sciences and Engineering, South China University of Technology, Wushan Road 381, Guangzhou 510641, China

Tel: (+86)20-22236819 ; E-mail: 201520120593@mail.scut.edu.cn; Fax: (+86)20-87112853

*Dr. Guanglei Zhao, corresponding authors

State Key Laboratory of Pulp and Paper Engineering, South China University of Technology, Guangzhou, Guangdong, 510641, China

Tel: (+86)20-87111770; E-mail:glzhao@scut.edu.cn(G.L.Zhao); Fax: (+86)20-87111770

Dr. Wu Hui

School of Food Sciences and Engineering, South China University of Technology, Wushan Road 381, Guangzhou 510641, China

Tel: (+86)20-87112373; E-mail: fehwu@scut.edu.cn; Fax: (+86)20-87112373

Dr. Yigang Yu

School of Food Sciences and Engineering, South China University of Technology, Wushan Road 381, Guangzhou 510641, China

Tel: (+86)20-22236819; E-mail: yuyigang@scut.edu.cn; Fax: (+86)20-87112853

Dr. Furao Lai,

School of Food Sciences and Engineering, South China University of Technology, Wushan Road 381, Guangzhou 510641, China

Tel: (+86)20-87112373; E-mail: felai@scut.edu.cn; Fax: (+86)20-87112373

**Dr. Xinglong Xiao, corresponding authors

School of Food Sciences and Engineering, South China University of Technology, Wushan Road 381, Guangzhou 510640, China

Tel: (+86)20-22236819; E-mail: hxiao@unb.ca ; Fax: (+86)20-87112853

HPLC results

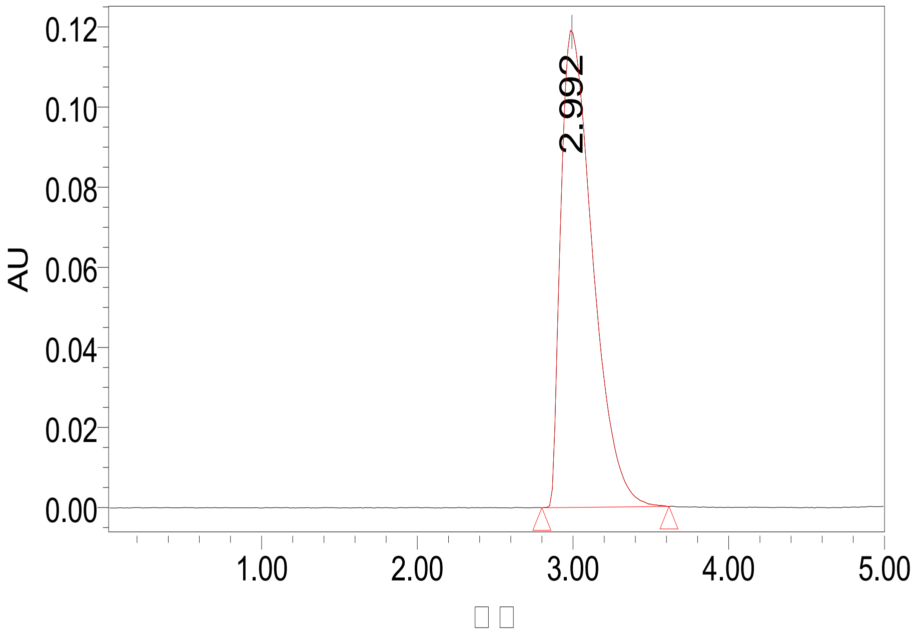

Fig. 1. Liquid chromatogram of arbutin before reaction.

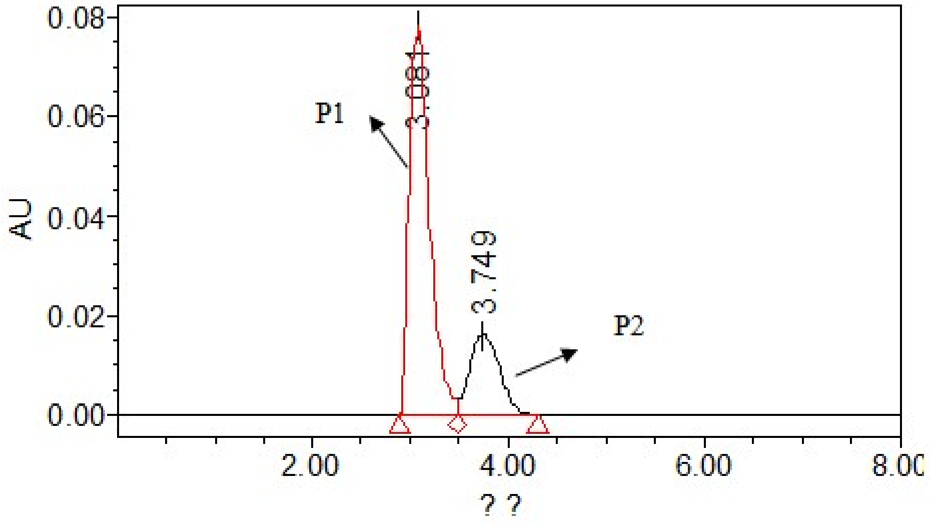

Fig. 2. Liquid chromatogram of the reaction at 1h.

P1: the peak of arbutin   P2: the peak of monoester

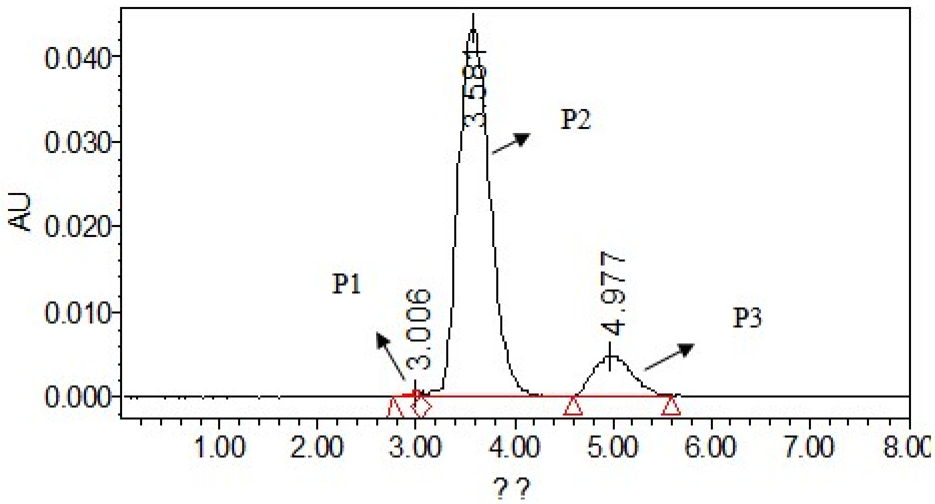

Fig. 3. Liquid chromatogram of the reaction at 24h.

P1: the peak of arbutin P2: the peak of monoester P3: the peak of diester
